# The triclinic form of dipotassium cobalt(II) bis­(dihydrogendiphosphate) dihydrate

**DOI:** 10.1107/S1600536811016345

**Published:** 2011-05-20

**Authors:** Abdellatif Lamhamdi, Rachid Essehli, Brahim El Bali, Michal Dušek, Karla Fejfarová

**Affiliations:** a Laboratory of Mineral Solid and Analytical Chemistry (LCSMA), Department of Chemistry, Faculty of Sciences, University Mohamed I, PO Box 717, 60000 Oujda, Morocco; b Institute of Physics of the ASCR, v.v.i., Na Slovance 2, 182 21 Praha 8, Czech Republic

## Abstract

In the title compound, K_2_Co(H_2_P_2_O_7_)_2_·2H_2_O, the octa­hedrally coordinated Co^2+^ ion lies on an inversion centre. Two bidentate dihydrogendiphosphate anions form the equatorial plane of the [CoO_6_] octa­hedron which is completed by two water mol­ecules in axial positions. This results in isolated {Co(H_2_O)_2_[H_2_P_2_O_7_]_2_}^4−^ entities linked into a three-dimensional network through K—O bonds and O—H⋯O hydrogen-bonding inter­actions involving the dihydrogendiphosphate anions and water mol­ecules. The dihydrogendiphosphate anion, (H_2_P_2_O_7_)^2−^, is bent and shows an almost eclipsed conformation.

## Related literature

The triclinic title compound is isotypic with K_2_Ni(H_2_P_2_O_7_)_2_·2H_2_O (Tahiri *et al.*, 2004 ▶) and K_2_Zn(H_2_P_2_O_7_)_2_·2H_2_O (Tahiri *et al.*, 2003 ▶). For ortho­rhom­bic forms of crystals of this formula type, see: Tahiri *et al.* (2002 ▶); Essehli *et al.* (2005 ▶).

## Experimental

### 

#### Crystal data

K_2_Co(H_2_P_2_O_7_)_2_·2H_2_O
*M* *_r_* = 525.1
Triclinic, 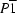

*a* = 6.8737 (14) Å
*b* = 7.3565 (11) Å
*c* = 7.6141 (14) Å
α = 80.740 (14)°
β = 72.397 (17)°
γ = 83.484 (14)°
*V* = 361.35 (12) Å^3^
*Z* = 1
Mo *K*α radiation
μ = 2.29 mm^−1^
*T* = 292 K
0.16 × 0.11 × 0.03 mm

#### Data collection

Oxford Diffraction Xcalibur diffractometer with a Sapphire 2 CCD detector
Absorption correction: analytical based on the crystal shape (*CrysAlis RED*; Oxford Diffraction, 2004 ▶) *T*_min_ = 0.648, *T*_max_ = 0.838
4546 measured reflections
1489 independent reflections
1013 reflections with *I* > 3σ(*I*)
*R*_int_ = 0.054

#### Refinement

*R*[*F* ^2^ > 2σ(*F* ^2^)] = 0.038
*wR*(*F* ^2^) = 0.090
*S* = 1.17
1489 reflections
118 parameters
Only H-atom coordinates refined
Δρ_max_ = 0.47 e Å^−3^
Δρ_min_ = −0.45 e Å^−3^

### 

Data collection: *CrysAlis CCD* (Oxford Diffraction, 2004 ▶); cell refinement: *CrysAlis RED* (Oxford Diffraction, 2004 ▶); data reduction: *CrysAlis RED*; program(s) used to solve structure: *SIR2002* (Burla *et al.*, 2003 ▶); program(s) used to refine structure: *JANA2006* (Petříček *et al.*, 2006 ▶); molecular graphics: *DIAMOND* (Brandenburg, 1999 ▶); software used to prepare material for publication: *JANA2006*.

## Supplementary Material

Crystal structure: contains datablocks global, I. DOI: 10.1107/S1600536811016345/wm2482sup1.cif

Structure factors: contains datablocks I. DOI: 10.1107/S1600536811016345/wm2482Isup2.hkl

Additional supplementary materials:  crystallographic information; 3D view; checkCIF report

## Figures and Tables

**Table:** 

K1—O2^i^	2.785 (3)
K1—O4^ii^	2.884 (3)
K1—O5	2.979 (3)
K1—O6^iii^	2.771 (4)
K1—O7^iv^	2.919 (3)
K1—O8^i^	2.972 (4)
Co1—O2	2.101 (3)
Co1—O5	2.085 (3)
Co1—O8	2.094 (3)
P1—O1	1.602 (3)
P1—O2	1.495 (3)
P1—O3	1.496 (3)
P1—O4	1.556 (3)
P2—O1	1.602 (3)
P2—O5	1.493 (3)
P2—O6	1.546 (3)
P2—O7	1.499 (3)

**Table:** 

P1—O1—P2	130.90 (19)

Symmetry codes: (i) 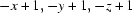; (ii) 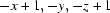; (iii) 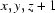; (iv) 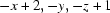.

**Table 2:** Hydrogen-bond geometry (Å, °)

*D*—H⋯*A*	*D*—H	H⋯*A*	*D*⋯*A*	*D*—H⋯*A*
O4—H1⋯O7^ii^	0.81 (5)	1.75 (5)	2.545 (5)	169 (5)
O6—H2⋯O3^v^	0.72 (6)	1.82 (6)	2.522 (5)	168 (5)
O8—H3⋯O3^vi^	0.71 (5)	2.03 (5)	2.745 (4)	178 (6)
O8—H4⋯O7^vii^	0.79 (5)	2.01 (6)	2.798 (5)	175 (5)

Symmetry codes: (ii) 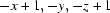; (v) 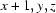; (vi) 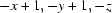; (vii) 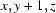.

**Fig. 1.**
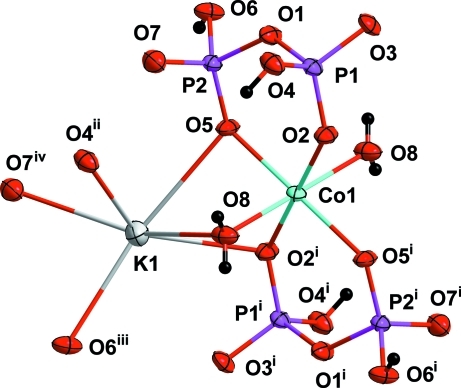
: The main coordination polyhedra in the title compound, shown as an ellipsoid plot with anisotropic displacement parameters drawn at the 50% probability level. [Symmetry codes: (i) 1 - x, 1 - y, 1 - z; (ii) 1 - x, -y, 1 - z; (iii) x, y, 1 + z; (iv) 2 - x, -y, 1 - z].

**Fig. 2.**
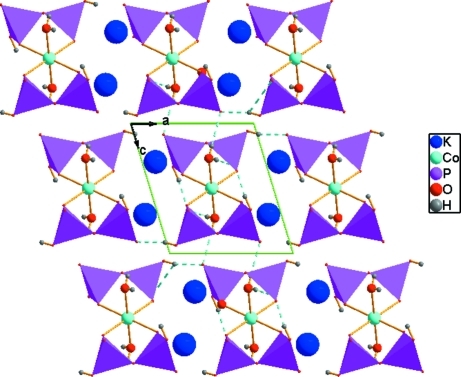
: Projection of the structure along the b-axis. H-bonds are displayed as dashed lines.

## supplementary crystallographic information

### Comment

The triclinic form of K_2_Co(H_2_P_2_O_7_)_2_^.^2H_2_O is isotypic with K_2_Ni(H_2_P_2_O_7_)_2_^.^2H_2_O (Tahiri *et al.*, 2004) and K_2_Zn(H_2_P_2_O_7_)_2_^.^2H_2_O (Tahiri *et al.*, 2003). All these K_2_*M*(H_2_P_2_O_7_)_2_^.^2H_2_O dihydrogendiphosphates crystallise also in an orthorhombic form, see: Tahiri *et al.* (2002) for *M* = Co; Essehli *et al.* (2005) for *M* = Ni and Zn.

The crystal structure of the title compound can be described in terms of centrosymmetric {Co(H_2_O)_2_[H_2_P_2_O_7_]_2_}^4-^ units (Fig. 1) that are linked through K—O bonds and an intricate network of O—H···O hydrogen bonds into a three-dimensional network (Fig. 2). The slightly distorted coordination octahedron around Co^2+^ is composed of four O atoms from two bidendate [H_2_P_2_O_7_]^2-^ groups in equatorial positions and two O atoms from water molecules in axial positions. The average Co—O bond length of 2.093 (8) Å is in the same range as 2.047 Å for the orthorhombic form (Tahiri *et al.*, 2002). The dihydrogendiphosphate anion is bent and shows an almost eclipsed conformation, with an bridging angle P1—O1—P2 of 130.90 (19) °. P—O bond lengths and O—P—O angles values are of similar values as in known dihydrogendiphosphates. The K^+^ cation is coordinated by six O atoms in form of a very distorted octahedron, with K—O distances ranging from 2.771 (4) to 2.979 (3) Å. Such values are likewise found in isotypic or isoformular dihydrogendiphosphates.

### Experimental

To prepare the present crystals we used the same procedure as described in detail in (Tahiri *et al.*, 2002). Solutions of CoCl_2_^.^4H_2_O (10 ml, 10 mmol) and K_4_P_2_O_7_ (10 ml, 20 mmol) were mixed in a beaker. The mixture was stirred for six hours and then allowed to stand for two weeks at room temperature. At the end of this period, large prismatic pink crystals have deposited, which were filtered-off and washed with a water-ethanol solution (20:80).

### Refinement

All hydrogen atoms were found in difference Fourier maps and their coordinates were refined independently. The isotropic atomic displacement parameters of hydrogen atoms were treated with 1.2×*U*_eq_ of the respective parent O atom.

### Crystal data

**Table:**

K_2_Co(H_2_P_2_O_7_)_2_·2H_2_O	*Z* = 1
*M**_r_* = 525.1	*F*(000) = 261
Triclinic, *P*1	*D*_x_ = 2.412 Mg m^−^^3^
Hall symbol: -P 1	Mo *K*α radiation, λ = 0.71069 Å
*a* = 6.8737 (14) Å	Cell parameters from 4546 reflections
*b* = 7.3565 (11) Å	θ = 3.1–26.6°
*c* = 7.6141 (14) Å	µ = 2.29 mm^−^^1^
α = 80.740 (14)°	*T* = 292 K
β = 72.397 (17)°	Prism, pink
γ = 83.484 (14)°	0.16 × 0.11 × 0.03 mm
*V* = 361.35 (12) Å^3^	

### Data collection

**Table:**

Oxford Diffraction Xcalibur diffractometer with a Sapphire 2 CCD detector	1489 independent reflections
Radiation source: Mo X-ray tube	1013 reflections with *I* > 3σ(*I*)
graphite	*R*_int_ = 0.054
Detector resolution: 8.3438 pixels mm^-1^	θ_max_ = 26.6°, θ_min_ = 3.1°
Rotation method data acquisition using ω scans	*h* = −8→8
Absorption correction: analytical based on the crystal shape (*CrysAlis RED*; Oxford Diffraction, 2004)	*k* = −9→9
*T*_min_ = 0.648, *T*_max_ = 0.838	*l* = −9→9
4546 measured reflections	

### Refinement

**Table:**

Refinement on *F*^2^	4 constraints
*R*[*F*^2^ > 2σ(*F*^2^)] = 0.038	Only H-atom coordinates refined
*wR*(*F*^2^) = 0.090	Weighting scheme based on measured s.u.'s *w* = 1/[σ^2^(*I*) + 0.0016*I*^2^]
*S* = 1.17	(Δ/σ)_max_ = 0.044
1489 reflections	Δρ_max_ = 0.47 e Å^−^^3^
118 parameters	Δρ_min_ = −0.45 e Å^−^^3^
0 restraints	

### Special details

**Table:**

Refinement. The hydrogen atoms were localized from the difference Fourier map. Their coordinates were refined independently. The isotropic temperature parameters of hydrogen atoms were calculated as 1.2**U*_eq_ of the parent atom.

### Fractional atomic coordinates and isotropic or equivalent isotropic displacement parameters (Å^2^)

**Table:**

	*x*	*y*	*z*	*U*_iso_*/*U*_eq_	
K1	0.89288 (17)	0.26574 (14)	0.70307 (15)	0.0365 (4)	
Co1	0.5	0.5	0.5	0.0172 (3)	
P1	0.32488 (16)	0.27153 (14)	0.24623 (14)	0.0181 (4)	
P2	0.75153 (16)	0.18965 (14)	0.24070 (14)	0.0177 (4)	
O1	0.5649 (4)	0.2309 (4)	0.1518 (4)	0.0242 (11)	
O2	0.2951 (4)	0.3930 (4)	0.3932 (4)	0.0213 (10)	
O3	0.2401 (4)	0.3486 (4)	0.0882 (4)	0.0241 (10)	
O4	0.2372 (5)	0.0812 (4)	0.3353 (4)	0.0263 (11)	
H1	0.252 (7)	0.045 (6)	0.437 (6)	0.0316*	
O5	0.7435 (4)	0.3267 (4)	0.3681 (4)	0.0231 (10)	
O6	0.9309 (4)	0.2143 (4)	0.0601 (4)	0.0258 (11)	
H2	1.011 (7)	0.252 (7)	0.083 (7)	0.0309*	
O7	0.7467 (5)	−0.0072 (4)	0.3310 (4)	0.0283 (11)	
O8	0.5496 (5)	0.7064 (4)	0.2702 (4)	0.0274 (12)	
H3	0.602 (8)	0.692 (7)	0.176 (7)	0.0329*	
H4	0.606 (7)	0.790 (7)	0.280 (7)	0.0329*	

### Atomic displacement parameters (Å^2^)

**Table:**

	*U*^11^	*U*^22^	*U*^33^	*U*^12^	*U*^13^	*U*^23^
K1	0.0433 (6)	0.0353 (6)	0.0362 (6)	0.0096 (5)	−0.0215 (5)	−0.0093 (5)
Co1	0.0201 (4)	0.0177 (4)	0.0175 (4)	0.0005 (3)	−0.0093 (3)	−0.0069 (3)
P1	0.0183 (5)	0.0215 (6)	0.0184 (5)	−0.0008 (4)	−0.0095 (4)	−0.0062 (4)
P2	0.0193 (5)	0.0204 (6)	0.0166 (5)	0.0009 (4)	−0.0084 (4)	−0.0068 (4)
O1	0.0183 (15)	0.0363 (18)	0.0229 (15)	0.0048 (12)	−0.0110 (12)	−0.0128 (13)
O2	0.0212 (15)	0.0236 (15)	0.0238 (15)	−0.0009 (12)	−0.0095 (12)	−0.0110 (12)
O3	0.0222 (16)	0.0337 (17)	0.0198 (15)	−0.0011 (13)	−0.0130 (13)	−0.0008 (13)
O4	0.0359 (18)	0.0235 (16)	0.0238 (17)	−0.0081 (13)	−0.0143 (15)	−0.0005 (13)
O5	0.0225 (16)	0.0294 (16)	0.0225 (15)	0.0037 (13)	−0.0099 (13)	−0.0156 (13)
O6	0.0215 (17)	0.0403 (19)	0.0180 (15)	−0.0081 (14)	−0.0039 (13)	−0.0105 (14)
O7	0.0388 (18)	0.0224 (16)	0.0269 (17)	−0.0040 (14)	−0.0137 (14)	−0.0024 (13)
O8	0.039 (2)	0.0253 (18)	0.0188 (16)	−0.0079 (15)	−0.0065 (15)	−0.0063 (14)

### Geometric parameters (Å, °)

**Table:**

K1—O2^i^	2.785 (3)	Co1—O8^i^	2.094 (3)
K1—O4^ii^	2.884 (3)	P1—O1	1.602 (3)
K1—O5	2.979 (3)	P1—O2	1.495 (3)
K1—O6^iii^	2.771 (4)	P1—O3	1.496 (3)
K1—O7^iv^	2.919 (3)	P1—O4	1.556 (3)
K1—O8^i^	2.972 (4)	P2—O1	1.602 (3)
Co1—O2	2.101 (3)	P2—O5	1.493 (3)
Co1—O2^i^	2.101 (3)	P2—O6	1.546 (3)
Co1—O5	2.085 (3)	P2—O7	1.499 (3)
Co1—O5^i^	2.085 (3)	O4—H1	0.81 (5)
Co1—O8	2.094 (3)	O6—H2	0.72 (6)
			
O2^i^—K1—O4^ii^	123.43 (10)	O5^i^—Co1—O5	180
O2^i^—K1—O5	60.33 (9)	O5^i^—Co1—O8	86.81 (11)
O2^i^—K1—O6^iii^	113.92 (10)	O5^i^—Co1—O8^i^	93.19 (11)
O2^i^—K1—O7^iv^	151.28 (8)	O8^i^—Co1—O8	180
O2^i^—K1—O8^i^	60.69 (9)	O1—P1—O2	109.27 (18)
O4^ii^—K1—O5	72.55 (9)	O1—P1—O3	105.02 (15)
O4^ii^—K1—O6^iii^	104.16 (10)	O1—P1—O4	106.45 (15)
O4^ii^—K1—O7^iv^	74.58 (9)	O2—P1—O3	116.17 (17)
O4^ii^—K1—O8^i^	68.68 (9)	O2—P1—O4	110.05 (16)
O5—K1—O6^iii^	166.01 (9)	O3—P1—O4	109.36 (19)
O5—K1—O7^iv^	113.42 (9)	O1—P2—O5	110.97 (16)
O5—K1—O8^i^	57.71 (8)	O1—P2—O6	98.96 (17)
O6^iii^—K1—O7^iv^	77.87 (9)	O1—P2—O7	107.47 (18)
O6^iii^—K1—O8^i^	108.32 (9)	O5—P2—O6	112.65 (18)
O7^iv^—K1—O8^i^	143.17 (10)	O5—P2—O7	114.33 (17)
O2—Co1—O2^i^	180	O6—P2—O7	111.27 (17)
O2—Co1—O5	92.27 (11)	P1—O1—P2	130.90 (19)
O2—Co1—O5^i^	87.73 (11)	K1^i^—O2—Co1	95.37 (10)
O2—Co1—O8	87.94 (13)	K1^i^—O2—P1	110.42 (15)
O2—Co1—O8^i^	92.06 (13)	Co1—O2—P1	132.80 (16)
O2^i^—Co1—O2	180	K1^ii^—O4—P1	150.05 (19)
O2^i^—Co1—O5	87.73 (11)	P1—O4—H1	114 (4)
O2^i^—Co1—O5^i^	92.27 (11)	K1—O5—Co1	90.21 (10)
O2^i^—Co1—O8	92.06 (13)	K1—O5—P2	127.17 (15)
O2^i^—Co1—O8^i^	87.94 (13)	Co1—O5—P2	130.12 (19)
O5—Co1—O5^i^	180	K1^v^—O6—P2	125.12 (18)
O5—Co1—O8	93.19 (11)	P2—O6—H2	107 (4)
O5—Co1—O8^i^	86.81 (11)	H3—O8—H4	101 (5)

Symmetry codes: (i) −*x*+1, −*y*+1, −*z*+1; (ii) −*x*+1, −*y*, −*z*+1; (iii) *x*, *y*, *z*+1; (iv) −*x*+2, −*y*, −*z*+1; (v) *x*, *y*, *z*−1.

### Hydrogen-bond geometry (Å, °)

**Table:**

*D*—H···*A*	*D*—H	H···*A*	*D*···*A*	*D*—H···*A*
O4—H1···O7^ii^	0.81 (5)	1.75 (5)	2.545 (5)	169 (5)
O6—H2···O3^vi^	0.72 (6)	1.82 (6)	2.522 (5)	168 (5)
O8—H3···O3^vii^	0.71 (5)	2.03 (5)	2.745 (4)	178 (6)
O8—H4···O7^viii^	0.79 (5)	2.01 (6)	2.798 (5)	175 (5)

Symmetry codes: (ii) −*x*+1, −*y*, −*z*+1; (vi) *x*+1, *y*, *z*; (vii) −*x*+1, −*y*+1, −*z*; (viii) *x*, *y*+1, *z*.
